# Therapeutic Potentials of Synapses after Traumatic Brain Injury: A Comprehensive Review

**DOI:** 10.1155/2017/4296075

**Published:** 2017-04-12

**Authors:** Zunjia Wen, Dong Li, Meifen Shen, Gang Chen

**Affiliations:** ^1^Department of Neurosurgery & Brain and Nerve Research Laboratory, The First Affiliated Hospital of Soochow University, 188 Shizi Street, Suzhou 215006, China; ^2^School of Nursing, Medical College of Soochow University, Suzhou 215006, China; ^3^Department of Neurosurgery, Lianyungang Hospital of Traditional Chinese Medicine, Lianyungang 222004, China

## Abstract

Massive studies have focused on the understanding of the pathobiology of cellular and molecular changes and injury mechanisms after traumatic brain injury (TBI), but very few studies have specially discussed the role of synapses in the context of TBI. This paper specifically highlights the role and therapeutic potentials of synapses after TBI. First, we review and conclude how synapses interact with constant structural, metabolic, neuroendocrine, and inflammatory mechanisms after TBI. Second, we briefly describe several key synaptic proteins involved in neuroplasticity, which may be novel neuronal targets for specific intervention. Third, we address therapeutic interventions in association with synapses after TBI. Finally, we concisely discuss the study gaps in the synapses after TBI, in hopes that this would provide more insights for future studies. Synapses play an important role in TBI; while the understandings on the synaptic participation in the treatments and prognosis of TBI are lacking, more studies in this area are warranted.

## 1. Introduction

It is well established that traumatic brain injury (TBI) is closely related to the occurrences, evolvements, and prognosis of psychiatric disorders, neuronal dysfunction, and cognitive impairment [1–3]; however, the mechanisms underlying the diseases at the cellular and molecular levels such as inflammation involvement, metabolic homeostasis imbalance, and synaptic injury remain elusive. If remain untouched, it may increase the potentials for many short-term (bleeding, headaches) and long-term (cognitive impairments) symptoms [2]; therefore, the studies on the mechanisms elaborating the cellular and molecular pathology should be put on the top agenda of TBI researches.

Synapse as a basic element for brain structure has been believed to play a significant role in the disadvantageous influences following TBI; the regular fusing of the synaptic vesicle and the plasma membrane and the orderly releasing of neurotransmitters into the synaptic cleft seem to be essential to the normal neuronal interaction [4]. Although it is now widely acknowledged that synapse is important to brain development and cognitive functions [5–7], the molecular mechanisms that the synapse structure and function changes induced by TBI remain largely unclear [8–10]. Besides, the databases on synapse categories have identified 109 domains involved in synaptic functions and more than 5000 synaptic proteins [11], yet very few of the synaptic proteins have been proven to be related with the synaptic dysfunction after TBI. Comprehensive reviews on the role of synapses after TBI are significantly necessary to figure out the study status and to elucidate future studies.

**Figure 1:**
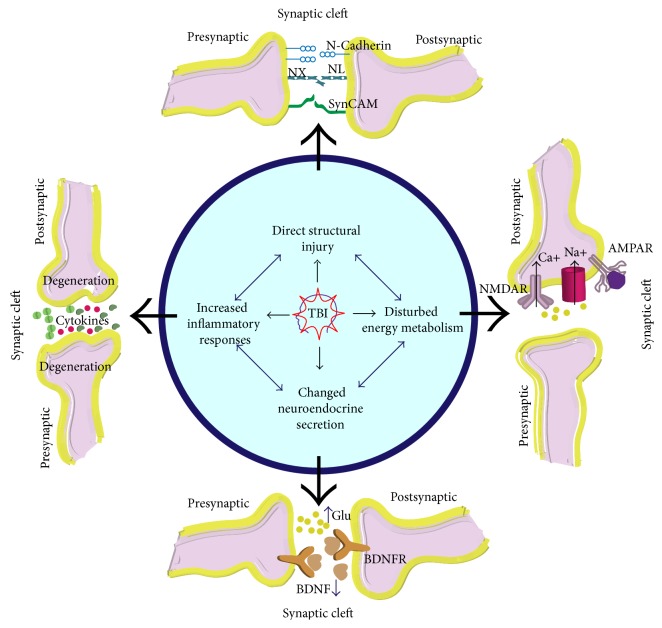
A brief drawing of the synaptic interaction with constant structural, metabolic, neuroendocrine, and inflammatory mechanisms after TBI; AMPAR: *α*-amino-3-hydroxy-5-methyl-4-isoxazolepropionic acid receptor; NMDAR: N-methyl-D-aspartate receptor; Glu: glutamate.

**Figure 2:**
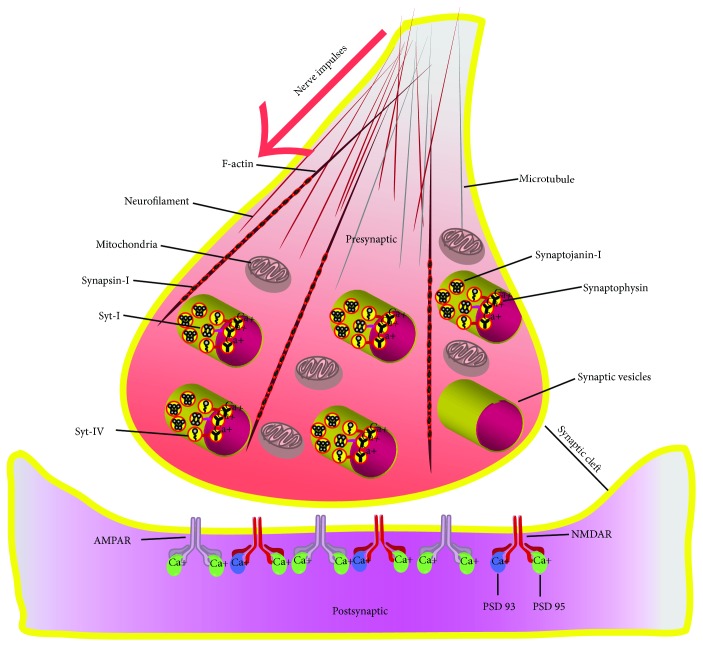
A brief distribution and construction of synaptic proteins. Notes: Syt-I: synaptotagmin-I; Syt-IV: synaptotagmin-IV; Syt-IV: synaptotagmin-IV; PSD-93: postsynaptic density complex protein-93; PSD-95: postsynaptic density complex protein-95.

TBI is a kind of disease with a lot of factors involved; the prognosis differs from one to another, and it is under the interaction of various mechanisms working unitedly or orderly, which make TBI treatment quite complicated. Therefore, we conducted this comprehensive review on the role of synapse after TBI, which mainly focused on the interactions between different functional mechanisms and synapses (Figure 1), the related synaptic proteins (Figure 2), and the targeted treatments on improving the synaptic plasticity after TBI, to provide insights into future studies in this area.

## 2. The Interactions between Functional Mechanisms and Synapses

### 2.1. The Interaction between Direct Structural Injury and Synapses after TBI

It is under estimation that more than 10^11^ neurons exist in the adult human brain, and each of them is constructed with 10^4^ synapses [12]. Synaptic structures have been reported to be highly vulnerable to the direct or indirect concussion attack following TBI in experimental and clinical settings [13, 14]. The focal mechanical attack resulting from the direct or contoured violence may lead to the structurally or functionally synaptic disconnection, and the structural changes of the synaptic cleft, the presynaptic and postsynaptic densities (PSDs), may cause temporary or long-term synaptic loss. This primary damages may trigger the biophysical and neurochemical change cascades and finally give rise to either synaptic repair or eternal loss.

### 2.2. The Interaction between Disturbed Energy Metabolism and Synapses after TBI

Membrane depolarization is initiated instantly by the damage on neuronal membranes and axons induced by TBI [15], leading to massively excitatory neurotransmitter release such as glutamate [16, 17]. The glutamate not only results in intracellular calcium accumulation but also activates the N-methyl-D-aspartate receptor (NMDAR) and *α*-amino-3-hydroxy-5-methyl-4-isoxazolepropionic acid receptor (AMPAR) [18, 19]. Additionally, mitochondrial dysfunction may occur due to the elevated calcium influx activating the intracellular proteases and finally lead to neuronal apoptosis. A huge body of energies are needed to maintain the metabolic and ionic homeostasis, and accordingly, the demand for blood glucose will increase, causing an imbalance between glucose supply and demand.

The synapses play a significant role in this process. Long-term potentiation (LTP) may be triggered by the activation of excitatory synapses, a process that requires intracellular calcium accumulation in the dendritic spine by activating NMDAR [20, 21]. The magnesium ions may block these receptor channels in a physiological situation with voltage-controlled method; however, in the stressing conditions induced by TBI, the cell depolarization largely by activating AMPAR may extrude the magnesium ions [22, 23]. Therefore, the NMDAR and AMPAR can significantly influence the LTP and eventually the energy metabolism.

### 2.3. The Interaction between Changed Neuroendocrine Secretion and Synapses after TBI

The disturbance of the hypothalamic-pituitary-adrenal (HPA) axis has been reported in several TBI studies [24–26]. The TBI may lead to increased serum cortisol and adrenocorticotropic hormone (ACTH) level to regulate other organs in this stressing condition; on one hand, those neuroendocrine hormones may alert the body to better deal with TBI, and on the other hand, the overexpression of stress hormones can significantly facilitate the TBI. However, it should be pointed out that although HPA axis changes after TBI is well documented, the direct linkages between synaptic dysfunction and HPA after TBI remain not clear, and the direct linkage of synaptic dysfunction to post-TBI HPA dysfunction needs to be further explored in future studies.

At the same time, it should be noted that the secretions of neurotrophins in the hippocampus are reduced after TBI. Neurotrophins served as “autocrine” in regulating the brain as the target organ contain many secretory proteins including brain-derived neurotrophic factor (BDNF), nerve growth factor (NGF), neurotrophin-3 (NT-3), and so on. Close relationships have been found between neurotrophins and synapse structures and functions; the neurotrophins may influence axonal and dendritic branching and remodelling [27–29], synaptogenesis in arborizing axon terminals [30], synaptic transmission [31–33], and synapsis maturation [32, 34]. In the context of TBI, the stress hormones are excessively secreted and neurotrophin secretion decreases; accordingly, the synapses tend to be seriously damaged. How to control and adjust the neuroendocrine secretion and synapsis development after TBI is a tricking yet promising problem in the treatment of TBI.

### 2.4. The Interaction between Increased Inflammation Responses and Synapses after TBI

TBI-induced physiological changes may give rise to neuroinflammation and neuron death [35]. A body of proinflammatory cytokines are rapidly elevated in the acute period including IL-6, TNF-*α*, and so on [36]; besides, the increased cytokines levels in the brain are dramatically higher than the corresponding levels in serum [37]. Generally, the existence of proinflammatory cytokines are required for preserving synaptic strength at excitatory synapses, and it is essential to synaptic plasticity [38, 39], but excessive secretion of proinflammatory cytokines may produce detrimental effects on the synapses [40].

The TBI-induced blood-brain barrier (BBB) integrity disruption acts an important role in the neuroinflammatory response [41], which allows increased cytokine pour into the brain and ultimately activate microglia in excess. The microglia is highly alerted in the acute period after TBI [41]. The microglia offers a beneficial hand to neuronal circuit formation via phagocytosing weak synapses and regulating neurogenesis [42], but excessive microglia accumulations may cause serious impairments to the synaptic pruning and disrupt the synaptic plasticity [43, 44]. It should be emphasized that microglia might be linked to synaptic integrity in the inflammation response, yet no studies have specifically dealt with this issue in the context of TBI. The direct evidence linking microglia activation and cytokine elevation of synaptic changes after TBI is lacking, more studies on these issue are warranted.

## 3. The Major Synaptic Proteins Involved in TBI

Up to date, more than 5000 synaptic proteins have been identified [11], and most of them have been found in association with neurological diseases such as stroke, TBI, Alzheimer's disease, and so on. Based on literature review, we identify several key synaptic proteins and introduce them briefly (Figure 2).

A linear polymer microfilament called F-actin, which is essential for such important cellular functions as the mobility and contraction of cells during cell division [45, 46], is considered to have structural polarity which is critical to synaptotagmins [47, 48]. Synaptotagmins family is a kind of calcium-binding protein located in the synaptic vesicles, and Syt-I and Syt-IV are the most relevant in TBI. Syt-I proteolysis may hinder the synaptic vesicle from docking to the presynaptic membrane terminal [49, 50]; besides, the accumulation of deformed Syt-I may cause disadvantageous effects on presynaptic function [51], while Syt-IV increases massively after TBI and tends to reduce synaptic activity [52]. The synapsins are a family of phosphoproteins with a function of regulating the release of neurotransmitter in the presynaptic area [53]. It has been hypothesized that increased oxidative stress after TBI may lead to synapsin-I loss and further disturb the interactions between synapses [54]. Synaptophysin is a kind of calcium-binding glycoprotein acting in vesicular trafficking, docking, synaptogenesis, and synaptic plasma membrane fusing in the presynaptic. It is noteworthy that the role of synaptophysin involved in TBI remains unclear; it is found that synaptophysin levels change differently between the mild and severe TBI [55], but another study addressing these issues remains somewhat contradictory [56]. Synaptojanins act a significant role in recycling vesicles at the presynaptic area. Synaptojanin-I is predominantly distributed in nerve terminals and is extremely sensitive to calpain digestion [57]; therefore, it has been taken as a novel target for degradomic calpain [51].

## 4. The Treatments Targeted on Synapses after TBI

With consideration to the important role synapses played in the TBI prognosis, lots of strategies have been adopted to enhance the synaptic plasticity and promote synaptic function, and even though the treatments may largely differ, the basic principles for speeding recovery from TBI remain similar, that is, promoting synaptogenesis and synaptic terminal reconnection and then exerting the neuroprotective effects [58].

Exercises are believed to be effective in improving TBI prognosis; the underlying mechanisms include changing the brain structural integrity by enhancing neurogenesis and angiogenesis with more secretions of growth factors promoting synaptic plasticity [59–61]. Notably, aerobic exercises such as tai chi and yoga have been popularly promoted for its potential advantages in healthy and ill-attacked populations [62–67]; however, the frequency and burden of exercise after TBI differ from one to another and remain to be further elucidated [68].

Several experiments in the animal with significant synaptic function improvement should be considered. Inhibiting endocannabinoid degradation may ameliorate the neurobehavioral, neuroinflammatory, and glutamate dyshomeostasis after TBI via reducing synaptic hyperexcitability [69]. Pycnogenol, one kind of bioflavonoid with significant antioxidant and anti-inflammatory properties, provides a beneficial effect on improving CA3-CA1 synaptic function in rats after TBI [70]. Another lab study [71] indicates that tyrosine kinase EphB3 produces deleterious effects on maintaining synaptic stability and plasticity after TBI. Resveratrol, a polyphenol compound with antioxidant properties, can upregulate synaptophysin and PSD 95 and suppress neuronal autophagy [72]. Rapamycin may exert suppressing effects on the neurogenesis and synaptic reorganization shortly after TBI in the dentate gyrus and cause a neuroprotective effect [73]. Additionally, dietary omega-3 fatty acids intake can protect against the decreased synaptic plasticity and impaired learning ability after TBI [74]. Meanwhile, the low-level laser therapy after TBI seem to increase the BDNF level and promote synaptogenesis [75]. However, these data only indicate the potential use for TBI treatments [76, 77], and more clinical evaluations are needed to assess the value of these findings.

## 5. Study Gaps and Future Direction

The understanding on the synaptic mechanisms involved in TBI still remains incomplete, and the interactions between synapses and the other injury mechanisms still need to be ascertained. Also, even though three-dimensional in vitro injury systems have been proposed to connect the injury degree and cell responses [78], the majority of studies are conducted in animals but not in humans, and verifying these findings in human use is a big step to move on. Particularly, the synaptic proteins in TBI are not well-studied; most studies are confined in the pathological conditions of stroke or subarachnoid hemorrhage, and more synaptic protein-related studies may facilitate the identification of new proteins and protein-targeted treatments.

Notably, the synapses seem to respond differently to the mild and severe TBI, indicating that subanalysis on the role of synapses in accordance with the degree of TBI is warranted, and based on the literature review, we found that the studies on the severe TBI were rather insufficient, regardless of molecular mechanisms or treatment options. In addition, it is important to take the dynamic characteristics of TBI into consideration; the synapses may act differently at different post-TBI periods. Besides, some studies conclude that combined therapies seem to exert synergistic effects and are more beneficial than single therapies [79, 80]; the role of synapse in this condition is not fully understood.

In conclusion, synapses play a significant part in the evolvement of TBI with a complicated link to various responsive mechanisms. With more acting mechanisms, synaptic protein treatments and synapsis-related treatments await to be elucidated, and further studies on this area are necessitated.
